# Effects of poly-γ-glutamic acid (γ-PGA) on plant growth and its distribution in a controlled plant-soil system

**DOI:** 10.1038/s41598-017-06248-2

**Published:** 2017-07-20

**Authors:** Lei Zhang, Xueming Yang, Decai Gao, Lingli Wang, Jie Li, Zhanbo Wei, Yuanliang Shi

**Affiliations:** 1 Institute of Applied Ecology, Chinese Academy of Sciences, 72 Wenhua Road, Shenhe District, Shenyang, Liaoning 110016 China; 2 University of Chinese Academy of Sciences, 19 Yuquan Road, Shijingshan, Beijing 100049 China; 3 Harrow Research and Development Centre, Agriculture and Agri-Food Canada, 2585 County Road 20, Harrow, Ontario N0R 1G0 Canada

## Abstract

To demonstrate the responses of plant (Pakchoi) and soil to poly-γ-glutamic acid (γ-PGA) is essential to better understand the pathways of the promotional effect of γ-PGA on plant growth. In this study, the effects of γ-PGA on soil nutrient availability, plant nutrient uptake ability, plant metabolism and its distribution in a plant-soil system were tested using labeled γ-PGA synthesized from ^13^C_1_-^15^N-L-glutamic acid (L-Glu). γ-PGA significantly improved plant uptake of nitrogen (N), phosphorus (P), and potassium (K) and hence increased plant biomass. γ-PGA greatly strengthened the plant nutrient uptake capacity through enhancing both root biomass and activity. γ-PGA affected carbon (C) and N metabolism in plant which was evidenced with increased soluble sugar contents and decreased nitrate and free amino acids contents. About 26.5% of the γ-PGA-N uptake during the first 24 h, after γ-PGA application, was in the form of intact organic molecular. At plant harvest, 29.7% and 59.4% of γ-PGA-^15^N was recovered in plant and soil, respectively, with a 5.64% of plant N nutrition being derived from γ-PGA-N. The improved plant nutrient uptake capacity and soil nutrient availability by γ-PGA may partly explain the promotional effect of γ-PGA, however, the underlying reason may be closely related to L-Glu.

## Introduction

γ-PGA is a biosynthetic anionic homo-polyamide consisting of D/L-glutamic acid (D/L-Glu) units connected by amide linkages between α-amine and γ-carboxylic acid groups^1, 2^. As a new environmental-friendly fertilizer synergist like poly-aspartate (TPA) and other poly-amino acids^3–5^, γ-PGA shows promising application potential in agricultural use for its anionic, biodegradable and biocompatible properties^6–9^. Studies showed that γ-PGA can significantly increase the production (in the vegetative growth stage) of cucumber^10^ and Chinese Cabbage^11^, and the production of wheat^12^ and rapeseed^13^ in both physiological and reproductive growth stage. Particularly, Xu *et al*.^11^ made an effort to reveal the promotional effect of γ-PGA on the growth of Chinese Cabbage from the perspective of plant N metabolism process. Here we try to explore the promotional effect of γ-PGA on plant growth from the perspectives of the availability, uptake and metabolism of nutrients in a plant-soil system.

The augmentation of plant production means more nutrients uptake by plant, which fundamentally depends on the nutrient availability in soil, nutrient acquisition and metabolism ability of plant^14^. Firstly, γ-PGA has been known to increase the plant N uptake and improve the soil N availability by enhancing microbial and urease activities^12, 13^. However, its impact on the availability and plant utilization of soil P and K nutrients are seldom involved. Secondly, root is an integral part of plant and plays the most important role in nutrients acquisition and efficiency^15^. Both the root size and activity determine the growth rate of shoot^16^. Studies observed that γ-PGA addition can improve the root biomass^10, 12^, however, the effect of γ-PGA on root activity has not been reported yet. Moreover, Xu *et al*.^11^ recently found that the N metabolism of Chinese Cabbage was significantly accelerated by γ-PGA. γ-PGA will be decomposed into low molecular mass matters (Glu molecular or polypeptide) when applied in soil and eventually mineralized into inorganic N and C^17^. Studies documented that Glu can be absorbed by plants in its molecular form^18^ and plays a key role in plant C and N metabolism^19, 20^. In this perspective, it is of great interest to ascertain whether the organic forms of γ-PGA can be directly adsorbed by plants as a metabolite and thus affect the plant C/N metabolism and growth. In addition, γ-PGA may also act as N and/or C source for supporting plant growth^21^, thereby it is worthy of exploring the turnover of γ-PGA in soil and the actual contribution of γ-PGA-N and -C to plant. Organic N compound, labeled with ^13^C and ^15^N, has been used to separate the direct uptake of intact amino acid molecule from the uptake of N from mineralized amino acid by plant^22–24^. Thus, it will enable us to uncover the potential metabolite and nutrition function of γ-PGA by applying dual-labeled γ-PGA synthesized from the ^13^C_1_, ^15^N-L-Glu to soil.

In this study, the effects of γ-PGA on soil N, P, K nutrients availability, plant nutrients uptake ability and plant C/N metabolism, and the organic uptake and nutrition contribution of γ-PGA to plant were investigated in a pot trial with application of ^13^C- and ^15^N-labeled γ-PGA. The objective of this study was to understand the responses of plant and soil to γ-PGA addition and γ-PGA’s distribution in plant-soil system.

## Results

### Response of plant and soil to γ-PGA

#### Plant biomass and nutrient uptake

**Figure 1:**
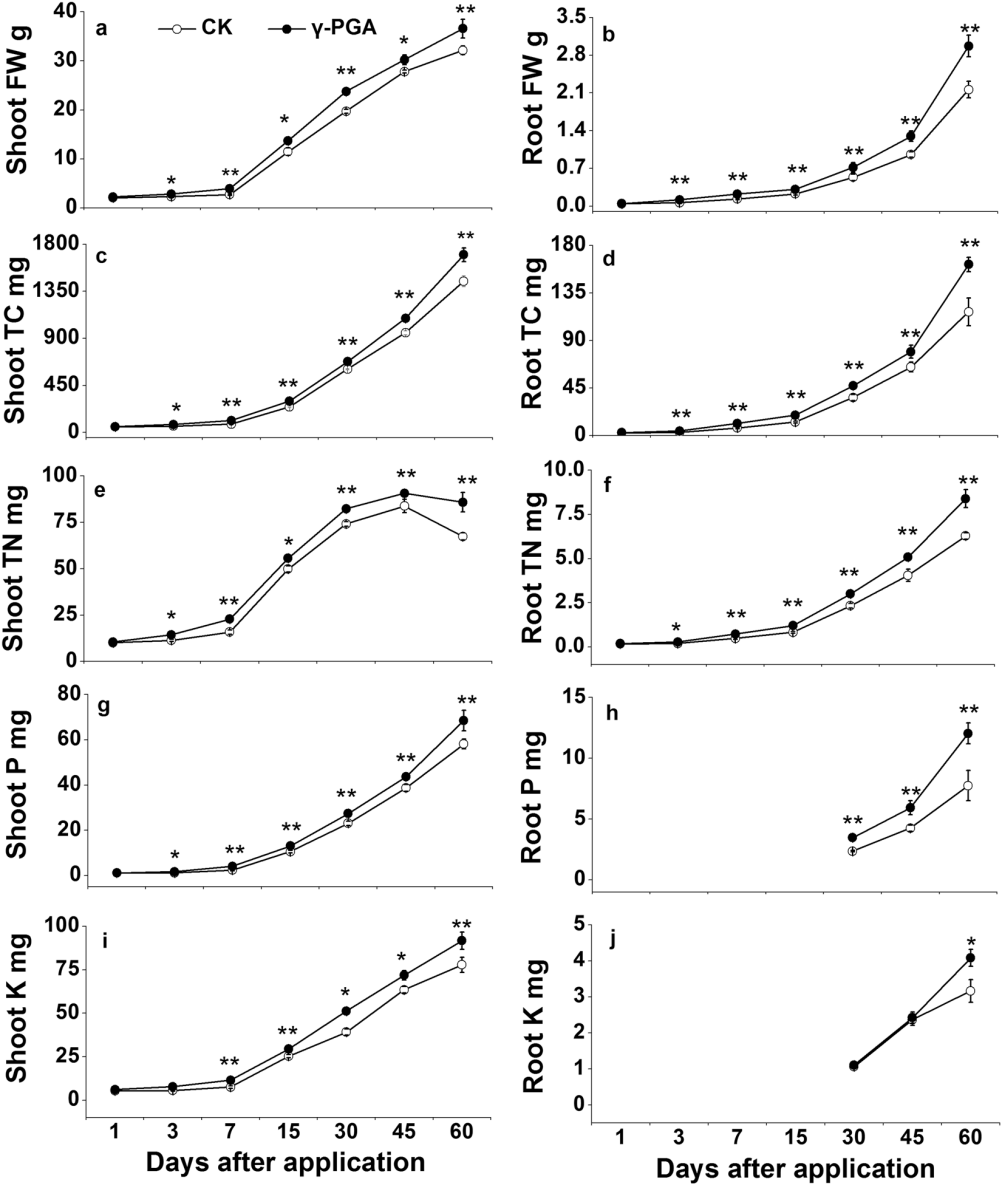
Temporal variations of fresh weight (FW, **a**,**b**), total carbon (TC, **c**,**d**), total nitrogen (TN, **e**,**f**), total phosphorus (TP, **g**,**h**) and total potassium (TK, **i**,**j**) in the shoot and root tissues, respectively, of the Pakchoi. Error bars represent standard error (n = 3). CK: Normal fertilization with no γ-PGA application, γ-PGA: Normal fertilization with application of 350.44 mg γ-PGA kg^−1^ soil. **p < 0.01, *p < 0.05.

Results showed that the significant differences in shoot and root fresh weight (FW) between the control (CK) and γ-PGA treatment were observed from day 3 to day 60 (p < 0.05, Fig. 1a,b). The FWs of shoot and root were pronouncedly enhanced by γ-PGA addition with increases of 26.7% and 47.4% at day 3, and 40.2% and 60.0% at day 7, respectively, in shoot and root, as compared with those of the CK treatment (p < 0.05). From day 15 to day 60, the FWs of shoot and root were 12.5–19.0% and 21.7–49.6% greater under γ-PGA treatment than under CK treatment, respectively (p < 0.05), indicating that the γ-PGA significantly promoted the plant growth with a larger growth rate in the early period (about one week after γ-PGA application).

Compared to the CK treatment, the γ-PGA treatment significantly enhanced the contents of total C (TC) and total N (TN) in shoot and root by 11.8–43.5% (shoot TC), 22.2–60.9% (root TC), 8.2–43.7% (shoot TN) and 25.4–51.8% (root TN), respectively, from day 3 to day 60 (p < 0.05, Fig. 1c–f). The total P (TP) contents in shoots were distinctly 12.6–45.8% greater in the γ-PGA treatment than in the CK treatment after day 3 (p < 0.05, Fig. 1g) and the TP contents in root were 39.6–55.4% larger in the γ-PGA treatment than in the CK treatment from day 30 to day 60 (p < 0.05, Fig. 1h). The total K (TK) contents in shoot were 13.1–51.5% higher in the γ-PGA treatment than in the CK treatment after day 7 (p < 0.05, Fig. 1i). Similarly, the use of γ-PGA resulted in 29.0% more TK in Pokchoi roots relative to the CK at day 60 (p < 0.05, Fig. 1j).

#### Plant C and N products and root activity (unlabeled γ-PGA)

**Figure 2:**
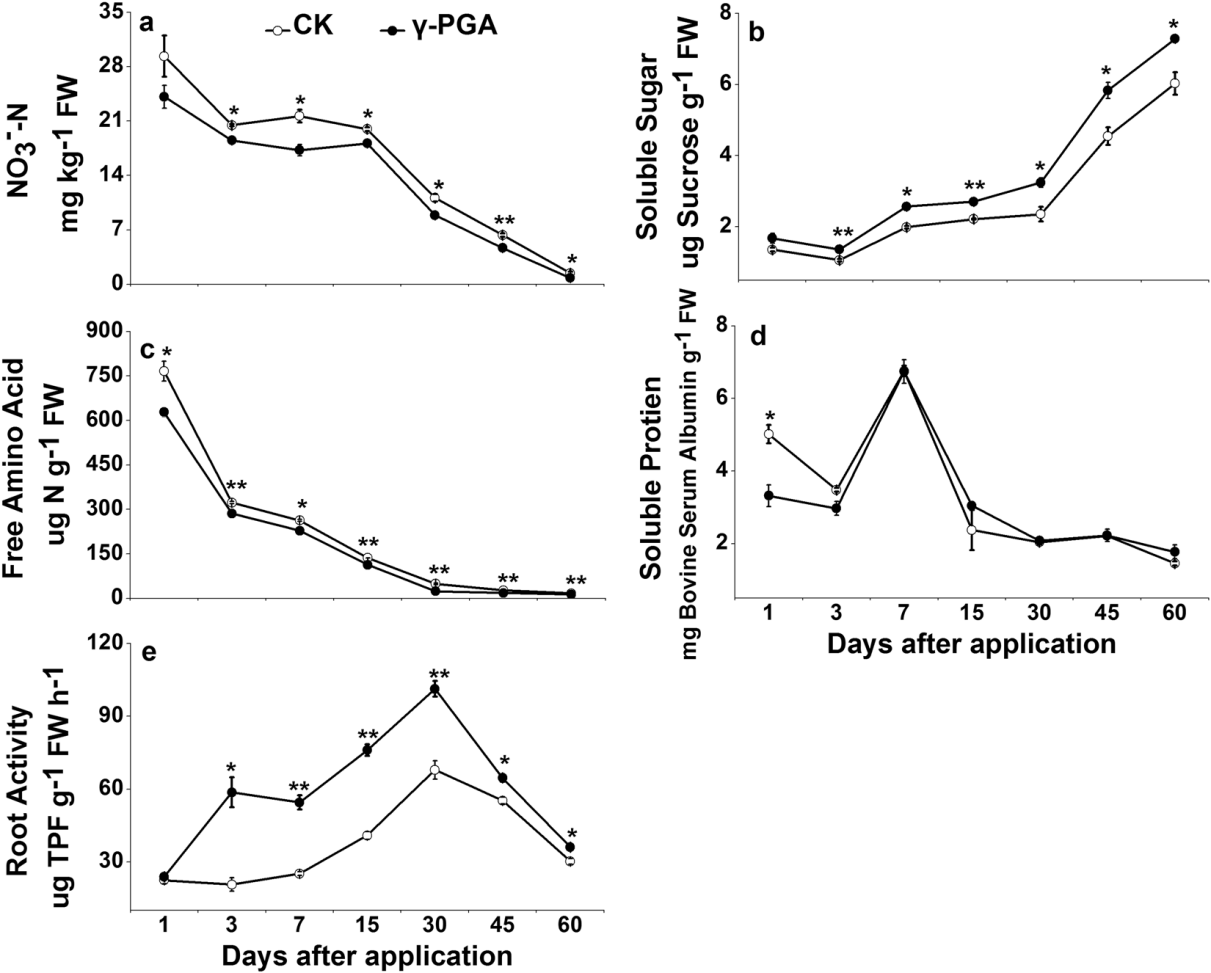
Temporal variations of shoot NO_3_^−^-N (**a**), soluble sugar (**b**), free amino acid (**c**) and soluble protein (**d**) and root activity (**e**) of the Pakchoi. Error bars represent standard error (n = 3). CK: Normal fertilization with no γ-PGA application, γ-PGA: Normal fertilization with application of 350.44 mg γ-PGA kg^−1^ soil. **p < 0.01, *p < 0.05.

Compared to the samples in the CK treatment, the nitrate (NO_3_^−^-N) contents in plant shoot were apparently lower by 9.6–42.3% in the γ-PGA treatment after day 3 (p < 0.05, Fig. 2a) and accompanying this the contents of free amino acids in plant shoot decreased in a similar pattern, 11.4–51.4% lower in the γ-PGA treatment after day 1 (p < 0.05, Fig. 2c). Soluble sugar contents in plant shoot kept rising as plant grew and its contents markedly raised by 20.8–37.8% in the γ-PGA treatment as compared with those in CK treatment (p < 0.05, Fig. 2b). γ-PGA showed little impact on the soluble protein content of plant shoot (Fig. 2d). Root activity gradually elevated as the plant grew and began to decline at day 30. The activity of roots were dramatically strengthened by 16.9–183.6% in the γ-PGA treatment in comparison to the CK treatment, from day 3 to day 30 (p < 0.05, Fig. 2e).

#### Soil nutrients availability

**Figure 3:**
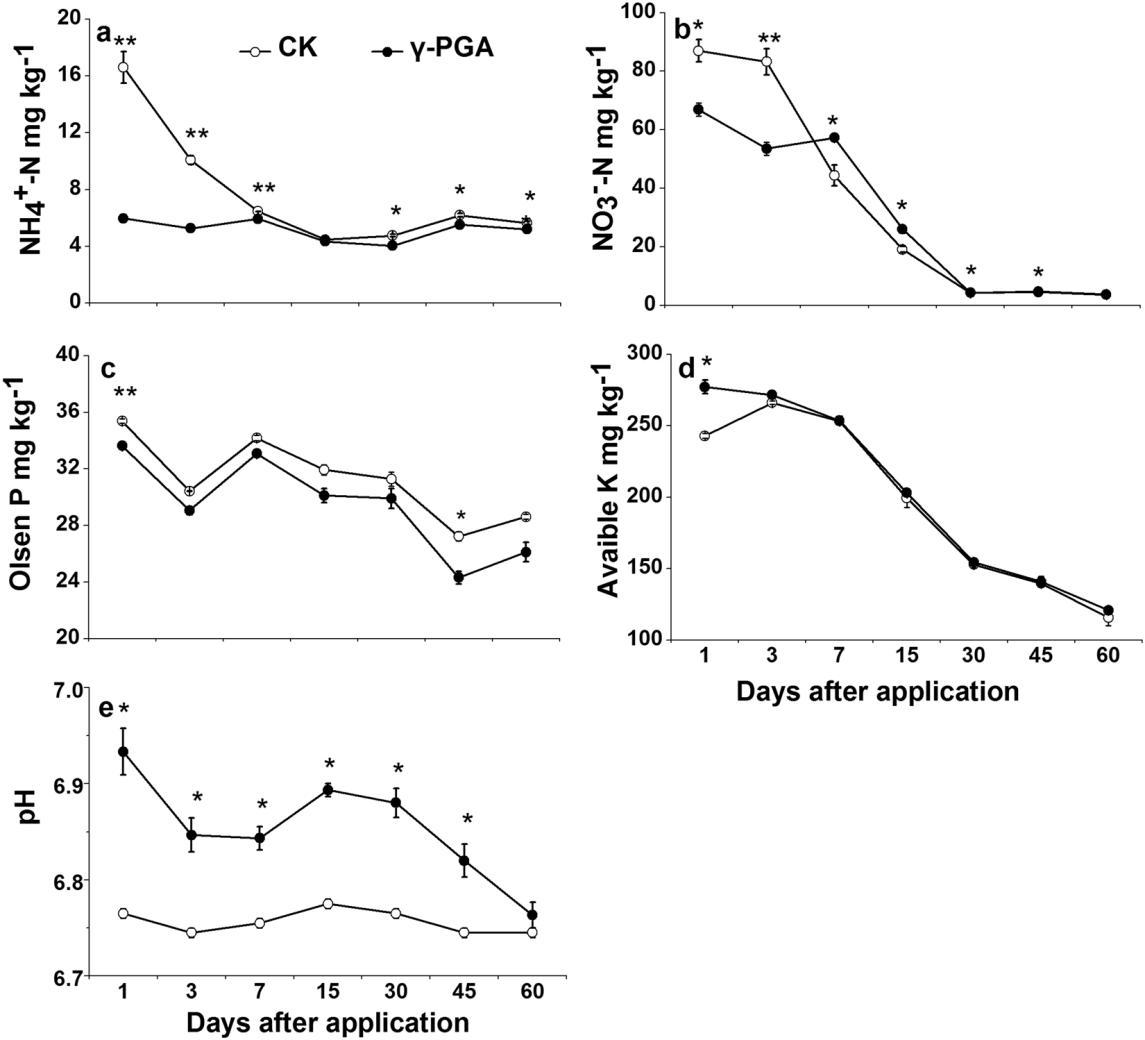
Temporal variations of soil NH_4_^+^-N (**a**), NO_3_^−^-N (**b**), Olsen-P (**c**), available K (**d**) content and pH (**e**). Error bars represent standard error (n = 3). CK: Normal fertilization with no γ-PGA application, γ-PGA: Normal fertilization with application of 350.44 mg γ-PGA kg^−1^ soil. **p < 0.01, *p < 0.05.

Soil ammonium (NH_4_^+^-N) concentrations were 7.9–64.0% less in the γ-PGA treatment than in the CK treatment during the whole study period, and the extent of this difference declined with time (p < 0.05, Fig. 3a). Soil NO_3_^−^-N contents were lower in the γ-PGA treatment than in the CK treatment at day 1 (23.12%) and day 3 (35.7%), whereas greater NO_3_^−^-N contents were found in the γ-PGA treatment than in the CK treatment after day 7 and the extent of difference declined from 36.3% at day 7, to 23.1% at day 15, to 5.0% at day 45 and 3.5% at day 60 (p < 0.05, Fig. 3b), respectively. Soil Olsen-P contents were 3.2–10.7% lower under the γ-PGA treatment than under the CK treatment (p < 0.05, Fig. 3c). Soil available K contents were not affected by γ-PGA addition during the entire study period (Fig. 3d). The use of γ-PGA significantly raised soil pH by 0.1 to 0.2 unit compared with the CK treatment from day 1 to day 45 (p < 0.05, Fig. 3e).

#### Soil microbial biomass and enzymatic activity

**Figure 4:**
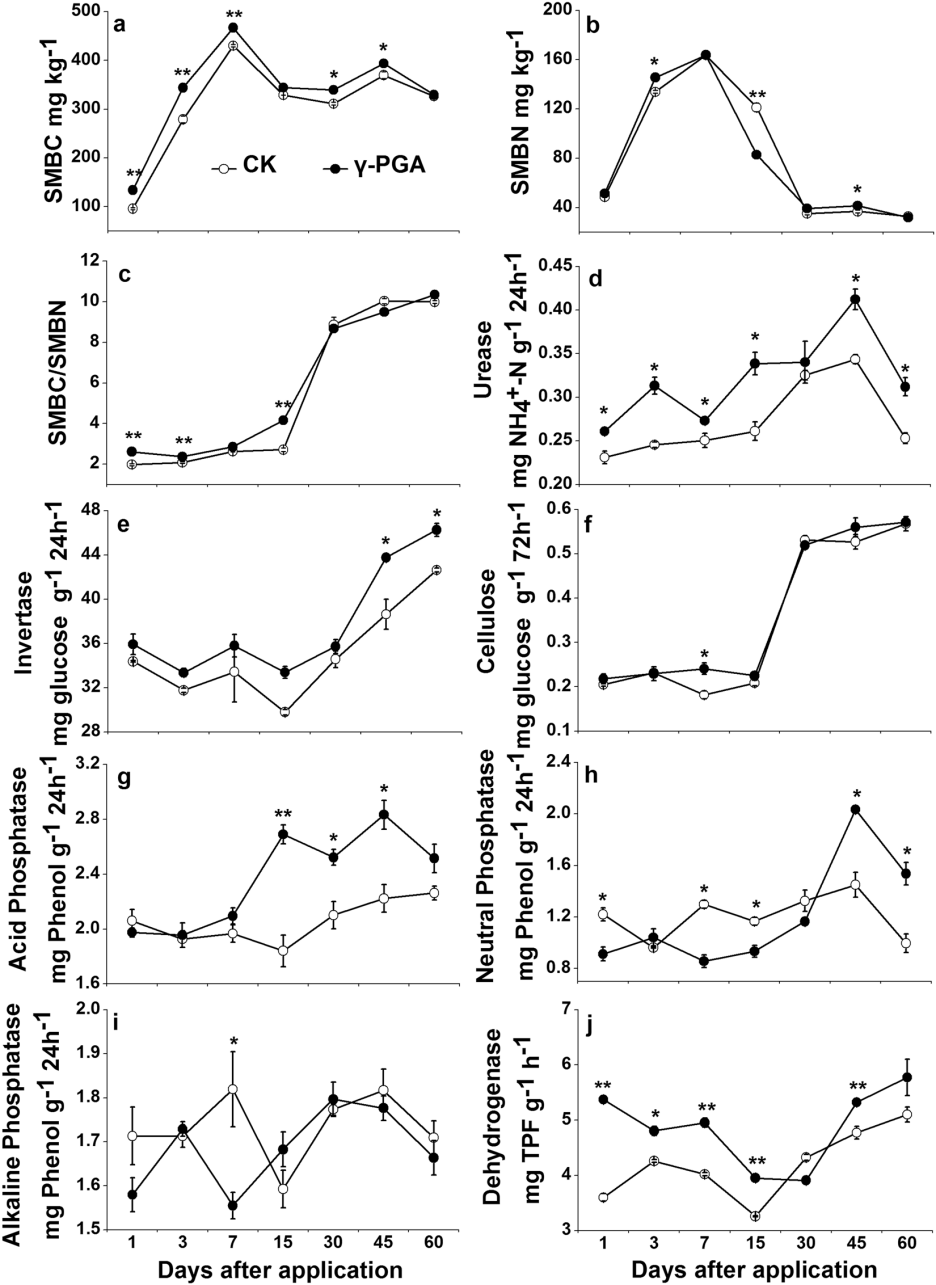
Temporal variations of soil microbial carbon (SMBC, **a**), soil microbial nitrogen (SMBN, **b**), SMBC/SMBN (**c**), soil urease (**d**), soil invertase (**e**), soil cellulose (**f**), soil acid phosphatase (**g**), soil neutral phosphatase (**h**), soil alkaline phosphatase (**i**) and soil dehydrogenase (**j**). Error bars represent standard error (n = 3). CK: Normal fertilization with no γ-PGA application, γ-PGA: Normal fertilization with application of 350.44 mg γ-PGA kg^−1^ soil. **p < 0.01, *p < 0.05.

The contents of soil microbial biomass C and N (SMBC and SMBN, varying from 96.1 mg kg^−1^ to 467.2 mg kg^−1^ and from 31.9 mg kg^−1^ to 163.8 mg kg^−1^, respectively) both reached the maximum at day 7, and then fell down to a constant level after day 30 (Fig. 4a,b). SMBC contents were obviously greater in the γ-PGA treatment than in the CK treatment at day 1 (39.6%) to day 45 (6.6%) (p < 0.05, Fig. 4a). SMBN contents under the γ-PGA treatment clearly improved by 12 mg kg^−1^ at day 3, whereas rapidly diminished to the same level as the CK treatment at day 30 and afterward (p < 0.05, Fig. 4b). The ratio of SMBC/SMBN was noticeably bigger under the γ-PGA treatment than under the CK treatment at day 1 (32.0%), day 3 (13.5%), day 15 (53.3%), however, there was no clear difference between treatments at the later period of this study (p < 0.05, Fig. 4c).

Soil urease activity was stimulated by γ-PGA addition showing 9.1–29.7% greater under the γ-PGA treatment than under the CK treatment (p < 0.05, Fig. 4d). Soil invertase activity was generally greater under the γ-PGA treatment than that under the CK treatment, but the difference was statistically significant only at day 45 (13.3%) and day 60 (8.5%) (p < 0.05, Fig. 4e). Soil cellulose activity was not significantly influenced by γ-PGA addition, except for day 7 (Fig. 4f). Soil acid phosphatase activity was greater (20.0–46.1%) under the γ-PGA treatment than under the CK treatment from day 15 to day 45 (p < 0.05, Fig. 4g). Neutral phosphatase activity in the soil treated with γ-PGA was significantly weaker before day 15, however, became higher at day 45 (40.3%) and day 60 (54.2%) as compared to that in the CK treatment (p < 0.05, Fig. 4h). There was no obvious difference in soil alkaline phosphatase activity between two treatments (Fig. 4i). Finally, the use of γ-PGA enhanced soil dehydrogenase activity, especially in the early period of γ-PGA addition, showing 11.6–49.2% higher in the γ-PGA treatment than in the CK treatment (p < 0.05, Fig. 4j).

### Distribution of γ-PGA in soil-plant system

#### Isotope abundance ratios (δ ^13^C and δ ^15^N)

**Figure 5:**
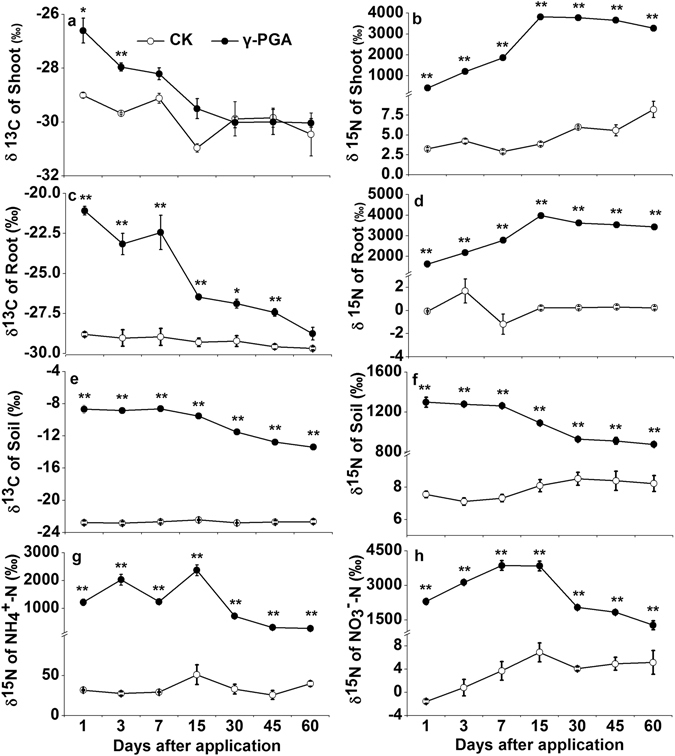
Temporal variations of δ^13^C in shoot (**a**), root (**c**), soil TC (**e**) and δ^15^N in shoot (**b**), root (**d**), soil TN (**f**), NH_4_^+^-N (**g**) and NO_3_^−^-N (**h**). Error bars represent standard error (n = 3). CK: Normal fertilization with no γ-PGA application, γ-PGA: Normal fertilization with application of 350.44 mg γ-PGA kg^−1^ soil. **p < 0.01, *p < 0.05.

Significant ^13^C and ^15^N enrichment were found in Pakchoi’s roots and shoots growed in the soil treated with ^13^C, ^15^N-γ-PGA (Fig. 5). The C stable isotope compositions (δ^13^C) of shoot and root tissues and soil all decreased as plant growth with a peak at day 1 (Fig. 5a,c,e). In comparison to those in the CK treatment, amending soil with ^13^C-^15^N-γ-PGA significantly increased the δ^13^C of plant shoot (−26.6‰ at day 1 and −27.96‰ at day 3) and plant root (changing from −21.1‰ to −27.4‰ before day 45), respectively, and also augmented the δ^13^C of soil samples (varying from −8.7‰ to −13.4‰ during the entire study period) (p < 0.05, Fig. 5a,c,e).

The N stable isotope compositions (δ^15^N) of plant shoot and root tissues went up rapidly from day 1 to day 15 and then kept a relatively constant level till the end of the study (Fig. 5b,d), and this was associated with the decline of the δ^15^N in soil samples (Fig. 5f). During the entire growth period of Pakchoi, the δ^15^N of shoot, root and soil samples were always significantly richer in the treatment amended with dual-labeled γ-PGA than that in the CK treatment (p < 0.05), varying between 415.6‰ and 3817‰ for the plant shoot, between 1630.3‰ and 3978.4‰ for the plant root and between 876.2‰ and 1297.7‰ for the soil sample, respectively (Fig. 5b,d,f). Both soil δNH_4_^+^-^15^N (ranging from 272.8‰ to 2368.1‰) and δNO_3_^−^-^15^N (ranging from 1272.0‰ to 3864.4‰) were very abundant at day 1 (1217.1‰ and 2304.2‰, respectively) and gradually increased to the crest on day 15 and then kept dropping till the harvest period (Fig. 5g,h). The statistical results showed that both δNH_4_^+^-^15^N and δNO_3_^−^-^15^N in the γ-PGA treatment were always more abundant than those in the CK treatment (p < 0.05).

#### Plant uptake of ^13^C and ^15^N labeled compounds

Ratio of ^13^C:^15^N in root was commonly used to estimate the fraction of N that was taken up in the form of intact amino acid^22–25^, because rapid transamination, deamination and decarboxylation of amino acids before allocation to shoots would make it difficult to recover amino acids in shoot tissue originating from direct uptake^26, 27^. In addition, two reasonable sampling periods for minizing the difference in molar ratio brought by sampling interval were suggested, which were 1–6 h and 24–72 h after tracer addition^14^. As a high-molecular-mass polymer, it takes time for γ-PGA to degrade into low-molecular-mass matter that can be directly absorbed by plant. So, 24 h was chosen as sampling time to calculate and assess the plant uptake of intact γ-PGA in this study.

Elevated ^13^C abundance in the root tissues indicated the uptake of labeled γ-PGA in the form of intact amino acid or polypeptide (Fig. 5b). Moreover, an elevated level of ^13^C abundance was also found in the shoots (Fig. 5a). The ratio of excess ^13^C to excess ^15^N (Ratio of ^13^C:^15^N) in the root was between 0.256 and 0.278 with an average of 0.265 (Table 1). Because each mole of the added L-^13^C_1_-^15^N-Glu incorporated one mole of ^13^C and one mole of ^15^N into γ-PGA, the ratio of ^13^C:^15^N corresponding to 100% uptake of intact amino acid is assumed to equal to 1. The average ^13^C:^15^N ratio of 0.265 meant that 26.5% of the N uptake was from intact amino acid (or polypeptide) at the day 1.

**Table 1:** ^13^C excess, ^15^N excess, and ^13^C:^15^N ratio in shoot and root of Pakchoi 24 h after γ-PGA application.

24 h	Root			Shoot		
	^13^C excess umol g^−1^ dry mass	^15^N excess umol g^−1^ dry mass	^13^C:^15^N	^13^C excess umol g^−1^ dry mass	^15^N excess umol g^−1^ dry mass	^13^C:^15^N
Sample 1	2.56	10.03	0.2557	0.71	8.48	0.0837
Sample 2	2.69	9.7	0.2775	0.82	7.04	0.1162
Sample 3	2.39	9.11	0.2622	0.87	6.5	0.1339
Average			0.2651			0.1113

#### Recoveries of γ-PGA-^13^C, -^15^N

Approximate 60.9% of the total γ-PGA-^13^C quickly degraded in the first day after the application of γ-PGA (Table 2). At day 60, 26.4% of γ-PGA-^13^C was recovered in soil-plant system, specifically 0.29% in plant and 26.1% in soil, respectively (Table 2). Total ^15^N recovery in the plant-soil system was 89.4% till the end of incubation (Table 3), with 27.2% and 2.79% in the root and shoot tissues and 59.4% in the soil, respectively (Table 3). In addition, the recoveries of NH_4_^+^-^15^N and NO_3_^−^-^15^N were much abundant before day 15 (ranging from 0.32% to 0.52% and from 4.85% to 10.67%, respectively), and then both rapidly dwindled after day 15 (ranging from 0.14% to 0.06% and from 0.44% to 0.23%, respectively).

**Table 2:** Recovery of γ-PGA-^13^C in the carbon (C) pools of plant-soil system at different sampling days.

Recovery of ^13^C (%)					
Day	Shoot	Root	Plant	Soil	Plant-Soil
1	0.050 ± 0.004a	0.0069 ± 0.001a	0.057 ± 0.004a	39.10 ± 1.32a	39.15 ± 1.32a
3	0.044 ± 0.003a	0.0084 ± 0.001a	0.052 ± 0.003a	38.80 ± 0.64a	38.85 ± 0.64a
7	0.037 ± 0.007a	0.025 ± 0.005ab	0.062 ± 0.004a	39.09 ± 0.95a	39.15 ± 0.94a
15	0.085 ± 0.009a	0.018 ± 0.001ab	0.10 ± 0.009a	35.78 ± 0.21b	35.88 ± 0.22b
30	0.102 ± 0.023a	0.035 ± 0.005bc	0.14 ± 0.018a	31.43 ± 0.26c	31.57 ± 0.27c
45	0.103 ± 0.054a	0.057 ± 0.006d	0.16 ± 0.059ab	27.99 ± 0.69d	28.15 ± 0.71d
60	0.241 ± 0.091b	0.047 ± 0.012cd	0.29 ± 0.103b	26.06 ± 0.98d	26.35 ± 1.04d

Means followed by the different letters in the same column are significantly different (p = 0.05). Plant ^13^C recovery was calculated as the sum of ^13^C recovery in shoot and root, ^13^C recovery of plant-soil was calculated as the sum of the ^13^C recovery in plant and soil.

**Table 3:** Recovery of γ-PGA-^15^N in the nitrogen (N) pools of plant-soil system at different sampling days.

Recovery of ^15^N (%)							
Day	Shoot	Root	Plant	NH_4_^+^-N	NO_3_^−^-N	Soil	Plant-Soil
1	0.46 ± 0.041a	0.026 ± 0.001a	0.49 ± 0.04a	0.32 ± 0.03b	7.69 ± 0.56b	98.70 ± 0.75a	99.19 ± 0.77a
3	1.61 ± 0.081b	0.057 ± 0.002a	1.67 ± 0.08b	0.52 ± 0.04a	8.18 ± 0.35b	93.73 ± 0.81b	95.40 ± 0.87b
7	3.83 ± 0.114c	0.19 ± 0.005b	4.02 ± 0.12c	0.34 ± 0.02b	10.67 ± 0.36a	90.92 ± 0.43b	94.94 ± 0.31b
15	20.76 ± 0.073de	0.45 ± 0.007c	21.21 ± 0.08d	0.44 ± 0.09ab	4.85 ± 0.45c	72.09 ± 1.25c	93.30 ± 1.23b
30	28.16 ± 0.116f	1.04 ± 0.017d	29.19 ± 0.10e	0.14 ± 0.004c	0.44 ± 0.02d	63.51 ± 0.74d	92.72 ± 0.81b
45	32.06 ± 0.472g	1.74 ± 0.035e	33.79 ± 0.44g	0.077 ± 0.003c	0.41 ± 0.01d	57.93 ± 0.73e	91.74 ± 0.92bc
60	27.18 ± 0.390e	2.79 ± 0.043f	29.69 ± 0.39f	0.059 ± 0.002c	0.23 ± 0.04d	59.40 ± 0.65e	89.37 ± 1.04c

Means followed by the different letters in the same column are significantly different (p = 0.05). Plant ^15^N recovery was calculated as the sum of ^15^N recovery in shoot and root, ^15^N recovery of plant-soil was calculated as the sum of the ^15^N recovery in plant and soil.

#### Contribution of γ-PGA-C, -N to plant biomass

γ-PGA-C contributed little to shoot-C, root-C and soil-C with a maximal input (0.07%, 0.20% and 0.37%, respectively) at day 1, only 0.011% of shoot-C, 0.023% of root-C and 0.24% of soil-C were derived from γ-PGA-C at the harvest period (Table 4). The contribution of γ-PGA-N to shoot-N and root-N both were peaked to 6.6% and 6.7% at day 15, respectively, and slowly declined with time to the final contributions of 5.6% and 5.9% at harvest, respectively (Table 5). The contribution of γ-PGA-N to soil N pool and plant-soil N pool both persistently declined with time, from 2.3% to 1.5% and from 2.3% to 2.0%, respectively. Soil NH_4_^+^-N and NO_3_^−^-N (derived from γ-PGA-N) ranged from 0.40–3.5% and 2.2–6.6%, respectively, during the entire 60 days (Table 5).

**Table 4:** Contributions of γ-PGA-C to the carbon (C) pools of plant-soil system at different sampling days.

C (%)					
Day	Shoot	Root	Plant	Soil	Plant-Soil
1	0.070 ± 0.004a	0.20 ± 0.007a	0.077 ± 0.004a	0.37 ± 0.013a	0.36 ± 0.013a
3	0.045 ± 0.004b	0.15 ± 0.017b	0.050 ± 0.003b	0.36 ± 0.005a	0.36 ± 0.005a
7	0.026 ± 0.005c	0.17 ± 0.028ab	0.039 ± 0.003c	0.37 ± 0.009a	0.36 ± 0.009a
15	0.022 ± 0.002cd	0.074 ± 0.004c	0.025 ± 0.002d	0.34 ± 0.002b	0.32 ± 0.002b
30	0.012 ± 0.003de	0.058 ± 0.008cd	0.015 ± 0.002e	0.29 ± 0.002c	0.27 ± 0.002c
45	0.007 ± 0.004e	0.056 ± 0.006cd	0.011 ± 0.004e	0.26 ± 0.006d	0.23 ± 0.005d
60	0.011 ± 0.004de	0.023 ± 0.006d	0.012 ± 0.004e	0.24 ± 0.010d	0.20 ± 0.009e

Means followed by the different letters in the same column are significantly different (p = 0.05); Contribution of γ-PGA-C to the C pool in plant-soil system was calculated as the percentage of the recovered γ-PGA-C to the total C in each C pool.

**Table 5:** Contributions of γ-PGA-N to the nitrogen (N) pools of plant-soil system at different sampling days.

N (%)							
Day	Shoot	Root	Plant	NH_4_^+^-N	NO_3_^−^-N	Soil	Plant-Soil
1	0.78 ± 0.065a	2.60 ± 0.079a	0.81 ± 0.064a	1.87 ± 0.15bc	4.06 ± 0.17c	2.30 ± 0.023a	2.28 ± 0.022a
3	1.98 ± 0.010b	3.65 ± 0.015b	2.02 ± 0.010b	3.52 ± 0.030a	5.42 ± 0.01b	2.22 ± 0.008b	2.21 ± 0.008ab
7	2.98 ± 0.019c	4.63 ± 0.094c	3.03 ± 0.020c	2.18 ± 0.07b	6.62 ± 0.36a	2.17 ± 0.025b	2.20 ± 0.024b
15	6.61 ± 0.063g	6.66 ± 0.036f	6.61 ± 0.062 g	3.54 ± 0.63a	6.58 ± 0.36a	1.87 ± 0.030c	2.24 ± 0.032ab
30	6.06 ± 0.030e	6.17 ± 0.179e	6.06 ± 0.023e	1.18 ± 0.02cd	3.62 ± 0.05cd	1.59 ± 0.009d	2.08 ± 0.008c
45	6.27 ± 0.123f	6.06 ± 0.081ed	6.26 ± 0.116f	0.49 ± 0.02d	3.23 ± 0.06d	1.51 ± 0.007e	2.09 ± 0.021c
60	5.61 ± 0.004d	5.88 ± 0.012d	5.64 ± 0.002d	0.40 ± 0.01d	2.19 ± 0.33e	1.50 ± 0.011e	1.99 ± 0.014d

Means followed by the different letters in the same column are significantly different (p = 0.05); Contribution of γ-PGA-N to the N pool in plant-soil system was calculated as the percentage of the recovered γ-PGA-N to the total N in each N pool.

## Discussion

Our results showed that plant fresh weight and C and N contents (in shoot and root tissues) were all significantly improved by the use of γ-PGA (Fig. 1), which is consistent with the findings of other studies^10–13^. Besides, we also found that γ-PGA could boost the plant uptake of P and K nutrients which was new in literature.

### Soil nutrient availability

Soil microbial biomass (SMBC and SMBN, Fig. 4a,b) and soil microbial activity (soil dehydrogenase activity, Fig. 4j) were both amplified by the application of γ-PGA. This is consistence with the finding that the soil respiration was remarkably enhanced after γ-PGA application^28^. Apparent augmentation in soil pH was also found in the γ-PGA treatment (Fig. 3e), which may be ascribed to (1) the poly-anionic nature of γ-PGA^6^ from the great amount of carboxyl attached to the main-chain of γ-PGA making the soil cation absorbed by γ-PGA and (2) the neutral charged γ-PGA solution applied to soil. These all indicate that amending soil with γ-PGA could stimulate biochemical changes in soil, which would exert deep effect on soil nutrient availability.

Soil urease activity was demonstrably activated by γ-PGA addition, which was similar to the results of Xu *et al*.^12, 13^. The NH_4_^+^-N content in soil treated with γ-PGA was greatly reduced, however, this may due to that more NH_4_^+^-N was adsorbed by γ-PGA and immobilized by enhanced microorganism biomass and plant growth. NO_3_^−^-N in soil is converted from NH_4_^+^-N under the function of nitrifying bacteria. Soil NO_3_^−^-N contents in γ-PGA treatment were significantly lower than that in the CK treatment within the first 3 days after γ-PGA application, which were consistent with the soil NH_4_^+^-N contents. However, after day 3 the soil NO_3_^−^-N contents became significantly higher in the γ-PGA treatment than that in the CK treatment, which may be attributed to the gradual oxidization of the NH_4_^+^-N released from γ-PGA surface and/or immobilization by enhanced microorganism biomass. This implies that γ-PGA is conducive to form NO_3_^−^-N at a delayed pace and would help to temporarily store more plant available N in soil. These stored mineral and microbial N would slowly release to provide crop N nutrient request somewhat in synchronicity to the crop’s needs^12, 13^. Soil acid and neutral phosphatase activities were also enhanced, which should be accompanied with more available P in soil at the late period of γ-PGA application. However, the soil Olsen P content was lower in the γ-PGA treatment than in the CK treatment. The presumed causes for this contradictory might be the microbial immobilization of available P by the enhanced soil microorganisms and the greater P nutrient uptake by Pakchoi after γ-PGA addition. There was almost no difference on the soil available K contents between treatments during the entire study period, which may be ascribed to the easy exchange of K^+^ between in soil solution and in soil mineral lattice. Clearly, soil nutrient availability was positively regulated by γ-PGA, especially the N nutrient.

### Plant nutrient uptake ability

Root biomass and activity were both significantly improved by γ-PGA in this study, and the promoted size and activity of plant root would have intensified plant nutrient uptake ability and elevated the plant growth^15, 16, 29^. Aside from its roles as metabolite and nutrition, several studies suggested that L-Glu can regulate plant growth by acting as a signaling molecule, especially to modulate the root development^30–32^. Walch-Liu *et al*.^33, 34^ found that external L-Glu, with a wide concentration range from 50 μM to 50 mM, could inhibite the primary root growth and encourage the lateral root branching of the *Arabidopsis*, which might lead to an increase in the density of the plant roots within glutamate-rich patches of soil^31, 34^. A similar response of plant root to L-Glu was found in other plant species, including *Thlaspi caerulescens*, *Thellungiella halophila*, *Papaver* (Iceland poppy) and *Solanum* (tomato)^33, 34^. The improved plant root biomass and activity in the γ-PGA treatment may be closely related to the degradation product of γ-PGA (L-Glu) in soil. A more elaborate study is needed to ascertain how γ-PGA affect the plant root growth and whether it will extend similar impact on plant root, like L-Glu does.

### Plant C and N metabolism and organic uptake of γ-PGA

The C and N metabolism of plant were clearly affected by γ-PGA with promoted soluble sugar contents, declined NO_3_^−^-N and free amino acids contents in plant grew in the soil amended with γ-PGA. These findings differed from the study of Xu *et al*.^11^ who noted that the contents of soluble protein and free amino acids in leaves of Chinese Cabbage were markedly enhanced by γ-PGA. We can not explain this descrepency except for speculate that it may result from different plant species and growth conditions. In this study, the ^13^C:^15^N ratio in the root tissue clearly showed that the intact L-Glu molecular or polypeptide uptake accounted for 26.5% of the total N uptake at day 1. In plants, Glu plays especially critical roles in C and N metabolism in company with glutamine synthase, glutamate synthase, glutamate dehydrogenase and aminotransferases^20, 31^. Some studies have demonstrated that root cells fed with L-Glu would elicit rapid cytosolic Ca^2+^ concentration increase and transient membrane depolarizations, which thus led to a series of changes in cytosolic ion concentration and finally influenced the process of plant metabolism^35–39^. Xu *et al*.^11^ also found that γ-PGA significantly improved the cytoplasmic free Ca^2+^ level and motivated the activity of Ca^2+^/CaM signaling pathway in plant cell, and thus accelerated the N metabolism of plant. The γ-PGA exhibited the similar physiological function on plant cell like L-Glu. We could speculate that the increasing efflux of L-Glu metabolite into the plant cell would cause a cascade of downstream responses to the plant C/N metabolism.

Multiple stepwise regression analysis showed a significant linear correlation between the shoot FW and the other variables: Y = 6.75 × X_1_ − 0.08 × X_2_ + 2.06 × X_3_ + 35.37 × X_4_ − 2.54 × X_5_ (R^2^ = 0.98, p < 0.001, where Y was the shoot FW, X_1_ was the root biomass, X_2_ was the root activity, X_3_ was the SMBC/SMBN, X_4_ was the urease activity and X_5_ was the dehydrogenase activity, respectively). Currently, we could deduce that γ-PGA may improve the plant growth by strengthening the uptake capacity of roots and regulating the nutrient availability through changing microbial and enzymatic characteristics. However, the crucial role of L-Glu in regulating both plant root growth and plant C/N metabolism implied that soil exogenous L-Glu decomposed from γ-PGA may be the underlying mechanism for γ-PGA to promote plant growth and nutrient uptake. Although the evidence for this hypothesis is still largely lacking, it is nevertheless an attractive concept that deserves further investigation.

### Distribution of γ-PGA in plant-soil system

The loss of 60.9% added γ-PGA-^13^C during the first 24 h suggested that the initial decarboxylation of the γ-PGA was rather rapid. Microbial metabolism resulted in quick decarboxylation of amino acids^26^, thus a majority of the γ-PGA-^13^C loss could be due to this process. Fokin *et al*.^40^ and Dippold & Kuzyakov^41^ also found that the carboxyl group of amino acids was more prone to respiratory decarboxylation than the other C atom. Furthermore, although Glu (one of the three acid amino acids in nature) had strong interactions with the soil solid phase^42^, it showed weaker affinity to soil solid phase than the neutral and alkine amino acids^43, 44^, and this to some extent enhanced its availibility to soil microbial organisms. There were still 26.1% of the total γ-PGA-^13^C recovered from soil even in 60 days after γ-PGA application, and this remained γ-PGA-^13^C in soil might had been incorporated into new microbial cell biomass or absorbed by soil soild phase^43, 44^.

Accumulation of γ-PGA-^15^N derived N in soil NH_4_^+^-N and NO_3_^−^-N increased rapidly in the first day after γ-PGA application, revealing that the mineralization of γ-PGA was rapid and happened immediately after the addition of γ-PGA. As the plant growth, the accumulation of γ-PGA-^15^N in plant sharply pyramided from 4.0% (day 7) to 21.2% (day 15), which was associated with a sharp drop of soil recovered NO_3_^−^-^15^N from 10.7% at day 7 to 4.9% at day 15. By the end of this study (day 60), the recovery rates of γ-PGA-^15^N reduced to about 59% in soil and added to about 30% in plant tissues with about 89% of γ-PGA-^15^N remained in the plant-soil system. We can infer that the γ-PGA-N transported from soil to plant continuously as the growth of plant and only 11% of the γ-PGA-N lost from the plant-soil system which is much less than the loss of γ-PGA-C (73% loss) in the same period. The reason for this big difference might be due to more CO_2_ loss than nitrogenous gases loss, which we did not measure.

## Conclusions

γ-PGA significantly increased plant yield and N, P, K nutrient uptake by strengthening the uptake capacity of roots and regulating the nutrient availability through changing microbial and enzymatic characteristics. The underlying mechanism on the promotion effect of γ-PGA might lie in L-Glu (structure unit and decomposition product in soil of γ-PGA). The intact organic molecular form of γ-PGA could be utilized by plant directly and γ-PGA could serve as N nutrient sources for plant. The positive role in plant growth and production and the high recovery rate of γ-PGA-N in plant-soil system suggested that γ-PGA could be an effective nitrogen fertilizer synergist and/or N fertilizer for agricultural use.

## Methods

### ^13^C, ^15^N-γ-PGA

γ-PGA can be produced inside the cells (as a composition of the capsule) or outside the cells (secreted into the incubation medium) by several *Bacillus* strains^45^ and the D/L-glutamic acid (D/L-Glu) incorporated into γ-PGA can be directly derived from the cultivation medium or through *de novo* synthesis of *Bacillus* strains^46^. γ-PGA used in this study were produced in an incubation medium with L-Glu existing in the medium. The γ-PGA labeled with ^13^C and ^15^N was synthesized using dual-labeled L-Glu at Liaoning Zhongke Bio-engineering Co., LTD and purified following the method described by Goto and Kunioka^47^. Dual-labeled L-Glu (L**-** ^13^C_1_-^15^N-Glu, 98 atom% ^15^N, 98 atom% ^13^C) was purchased from Sigma-Aldrich (Shanghai) Trading Co., Ltd., and ^13^C was only labeled to the first carbon of L-Glu, namely the carboxyl carbon attached to the main chain of γ-PGA. The isotope ratio of ^13^C and ^15^N (δ^13^C and δ^15^N) of purified γ-PGA were 2407.54‰ and 58952‰, respectively, about 21% of the L-Glu incorporated into γ-PGA was L**-** ^13^C_1_-^15^N-Glu. The sample of γ-PGA was stored under 4 °C until its application in solution forms. At the same time, the unlabeled γ-PGA was prepared with unlabeled L-Glu under the same condition and purified with the same procedure.

### Experimental design

A pot trial was conducted in greenhouse in the Institute of Applied Ecology, Chinese Academy of Sciences, Shenyang, China. Pot was sized in 11 cm diameter × 10 cm height, drainage holes in the bottom, placed on a fitted plastic tray. A sandy clay loam soil was collected from a cultivated field located at Ecological Experimental Station of Chinese Academy of Sciences Shenyang, China (41°32′N, 122°23′E) in November 2014. The soil is classified as aquic Brown soil by Chinese Soil Classification (equivalent to a Haplaqualf by USDA Soil Taxonomy) with the following characteristics: pH (H_2_O) 6.72, TC 17.32 g kg^−1^, TN 1.45 g kg^−1^, NO_3_^−^-N 72.23 mg kg^−1^, NH_4_^+^-N 3.25 mg kg^−1^, TP 0.48 g kg^−1^, Olsen P 32.44 mg kg^−1^, TK14.68 g kg^−1^, available K 185.66 mg kg^−1^, and 25.61%, 23.57%, and 50.83% of clay, silt and sand, respectively. The soil was air-dried, ground, sieved (<5-mm) and homogeneously mixed. Stones, plant residues and visible soil fauna were excluded.

The experiment lasted for 75 days from Jun. 29, 2015 to Sep. 12, 2015. There were three treatments in this study, including one amended with ^13^C, ^15^N-γ-PGA, one with unlabeled γ-PGA and a control (no γ-PGA addition). All three treatments received the same rate of fertilizer, including 342.7 mg CO(NH_2_)_2_ kg^−1^ soil, 118.4 mg Ca(H_2_PO_4_)_2_.H_2_O kg^−1^ soil, and 246.8 mg K_2_SO_4_ kg^−1^ soil, and in addition, the unlabeled and labeled γ-PGA treatments received 350.44 mg kg^−1^ soil unlabeled and labeled γ-PGA, respectively. The chemical fertilizers were homogeneously mixed with soil before the soil was packed into the pot. A total of 84 plots were prepared as replicates (3 replicates for γ-PGA treatments and 6 replicates for CK) for subsampling (7 times). All pots were randomly placed with a distance of half meter between 2 pots in a greenhouse and the unlabeled and labeled pots were separately placed with a distance of 5 meters.

Ten Pakchoi (*Brassica rapa subsp*. *chinensis*) seeds were planted in each pot, the same amount of distilled water was added to all pots every two days and the water content (w/w) was maintained about 10–15% until the end of trial. At the third leaf stage of Pakchoi (13 days after the seed planting, Jul. 12), seedlings in each pot were thinned to six plants, and these six seedlings were distributed as evenly as we could manage in a circle (4 cm diameter) of the pot. γ-PGA was applied into the pots two days after the Pakchoi seedlings were fixed to six plants (Jul. 14). Fifty ml of γ-PGA solution was firstly injected into six different locations of soil (each in the middle of two adjacent plants with a distance of 3.5 cm from the center of the pot) to spread them evenly throughout the soil. When injecting, the needle (length 10 cm, diameter 2 mm) was inserted into soil with an angle of 45° to the soil surface and the solution was inserted as evenly as possible as the needle was slowly removed from the soil^22^. To eliminate the influence of N in γ-PGA, a given amount of CO(NH_2_)_2_ equivalent to the N content of γ-PGA was added to the CK treatment in the same day of γ-PGA application.

### Sampling and analysis of soil and plant

#### Soil and plant sampling

A total of seven batches of soil and plant samplings were performed on the 1, 3, 7, 15, 30, 45, 60 day, respectively, after γ-PGA application (representing day 1, day 3, day 7, day 15, day 30, day 45, day 60). At each sampling time, 3 pots from each γ-PGA treatment and 6 pots from CK (every 3 pots were randomly chosen as the CK of labeled and non-labeled γ-PGA treatment, respectively) were carefully moved to the laboratory. In laboratory, the above-ground part (shoot) of Pakchoi was removed at ground level and the root was carefully collected. The shoot and root samples were cleaned with distilled water. In the labeled γ-PGA treatment, the cleaned root materials were soaked in a 0.05 M CaCl_2_ solution for two hours to remove residual ^15^N attached to the outer surface of the root, and then rinsed with distilled water. The shoots and roots were fresh weighed and immediately deactivated at 105 °C for 2 h and dried in 70 °C, and following that the dry weights were collected. Dry shoot and root samples were ball milled to fine powder for further analysis of δ^13^C, δ^15^N and TN, TC, TP, TK contents. The soil in the pot was thoroughly homogenized and one sub-set of this soil was stored in the 4 °C for the analyses of soil NH_4_^+^-N, NO_3_^−^-N, SMBC and SMBN contents, and enzyme activities in 24 h and a second sub-set of this sample was air-dried and ground for the determination of δ^13^C, δ^15^N, and TN, TC, Olsen-P, and available K content. δ^13^C and δ^15^N of plant and soil samples in CK treatments were used to provide natural abundance date of δ^13^C and δ^15^N, allowing excess amounts of the isotopes in the treatments with dual-labeled γ-PGA to be calculated. In the non-labeled γ-PGA treatment, the shoots were immediately ground to homogenate at 0 °C for the determination of plant NO_3_^−^-N, soluble sugar, free amino acid, and soluble protein contents. The roots materials were used to measure the root activity.

#### Analysis of soil and plant samples

The concentrations of TN and TC in soil and plant were analyzed using an Elemental Analyzer (Vario MACRO Cube). Soil NH_4_^+^-N and NO_3_^−^-N were extracted with 2 M KCl solution (fresh soil:2 M KCl = 1:5, shaking for 1 h) and the ^15^N abundance of soil NH_4_^+^-N and NO_3_^−^-N were evaluated using ammonium diffusion method^48, 49^. The δ^13^C of plant and soil TC, and the δ^15^N of plant and soil TN, soil NH_4_^+^-N and NO_3_^−^-N samples were measured by an Elementary Analyzer (Thermo-Element Flash EA 1112, USA) coupled with an Isotope Ratio Mass Spectrometer (Thermo Fisher MAT 253, USA). Soil Olsen-P, soil available K, plant TP and plant TK were analyzed based on methods of Lu^50^ (the amount of root samples collected before day 30 were not enough for the determination of TP and TK contents). Soil pH was assayed (1:2.5 soil to solution ratio) using an automatic potentiometric titrator (ZD-2A, Shanghai, China). Soil gravimetric water content was measured by drying soil at 105 °C for 24 h. Soil texture was evaluated using the pipette-sedimentation method^51^.

Free amino acid content in plant was estimated following the Ninhydrin colorimetric method^52^. Plant soluble protein content was evaluated using the Coomassie brilliant blue G-250 dyeing method^53^. The NO_3_^−^-N content in plant was determined following the Salicylic Acid colorimetric method, plant soluble sugar content was measured with the Anthrone colorimetric method and root activity was assayed as the 2,3,5-Triphenyltetrazolium chloride (TTC) method, respectively^54^.

SMBC and SMBN were determined using the chloroform fumigation extraction method^55, 56^. Soil enzymatic activities including urease, invertase and cellulose activities related to C and N cycles, acid, neutral and alkaline phosphatase activities related to P cycle, dehydrogenase activity represented overall soil microbial activity^57^ were analyzed. In detail, soil urease activity was determined with the Phenol-hypochlorite colorimetric method^58, 59^. Invertase activity and cellulose activity were assayed with the method of 3, 5 dinitrosalicylic acid colorimetry as described by Frankenberger Jr & Johanson^60^ and Miller *et al*.^61^, respectively. The measurement of acid, neutral and alkaline phosphatase activity and dehydrogenase activity were performed based on the methods of Tabatabai^62^.

### Statistical analyses

All data sets were expressed as a mean of three replicates with a standard error. Differences on the plant and soil variables between the CK treatment and γ-PGA treatment were analyzed with one-way analysis of variance (ANOVA). Statistical significance was accepted at the p = 0.05 level. Multiply stepwise linear regression correlation was used to analyze the relationship between the shoot biomass and the other variables. The SPSS 19.0 software package (SPSS Inc., IL, USA) was used for statistical analyses.
